# Anti-Ro52/TRIM21 serological subsets identify differential clinical and laboratory parameters

**DOI:** 10.1007/s10067-022-06299-5

**Published:** 2022-07-23

**Authors:** Adrian Y. S. Lee, Ming-Wei Lin, Joanne H. Reed

**Affiliations:** 1 Westmead Institute for Medical Research, The University of Sydney, Westmead, NSW Australia; 2 Westmead Clinical School, The University of Sydney, Westmead, NSW Australia; 3 Department of Clinical Immunology, Westmead Hospital, Hawkesbury Road, Westmead, NSW 2145 Australia; 4 Department of Immunopathology, ICPMR and NSW Health Pathology, Westmead Hospital, Westmead, NSW Australia; 5 School of Medical Sciences, Faculty of Medicine and Health, The University of Sydney, Camperdown, NSW Australia

**Keywords:** Anti-La, Anti-Ro52/TRIM21, Anti-Ro60, Autoantibodies, Autoimmunity, Line immunoassay

## Abstract

**Introduction:**

Anti-Ro52/tripartite motif-containing protein 21 (TRIM21) IgG is one of the most common autoantibodies found in systemic autoimmune diseases and is typically found in conjunction with anti-Ro60 and/or anti-La. A retrospective, cross-sectional study was undertaken to examine the clinical and laboratory features of two serological subsets: patients with anti-Ro52/TRIM21 autoantibodies in the absence of anti-Ro60 and anti-La (isolated anti-Ro52/TRIM21) and patients with anti-Ro52/TRIM21 in the presence of anti-Ro60 and/or anti-La.

**Methods:**

Over a 12-month period, patients tested positive for anti-Ro52/TRIM21 via line immunoassay (LIA) at the Westmead Hospital (Australia) immunopathology laboratory were included. The presence of anti-Ro60 and/or anti-La via same LIA was noted. Associated laboratory and medical records were perused to extract demographic, laboratory, and clinical information.

**Results:**

There were 346 patients within the study period, and 39.9% of the patients positive for anti-Ro52/TRIM21 lacked anti-Ro60/anti-La autoantibodies. Isolated anti-Ro52/TRIM21 patients tend to be older, have lower anti-Ro52/TRIM21 titres, have lower rheumatoid factors, and have lower proportions of neutropaenia compared to patients who were positive for anti-Ro52/TRIM21 and anti-Ro60/La. This occurred independent to diagnoses of Sjögren’s syndrome or systemic lupus erythematosus. Coexisting neurological syndromes, pulmonary pathologies, and malignancies were more prevalent in the isolated anti-Ro52/TRIM21 subset.

**Conclusions:**

Patients with isolated anti-Ro52/TRIM21 tend to have distinct and important clinical and laboratory associations. It is unclear if these patients evolve or remain a stable subset and how they originate immunologically. Longitudinal and prospective studies are required to ascertain the overall predictive and prognostic value of this stratification.

**Table:** 

**Key Points** *• Anti-Ro52/TRIM21 is an autoantibody found in autoimmunity and non-immunological conditions.* *• Sixty percent of anti-Ro52/TRIM21 patients are positive for anti-Ro60.* *• Isolated anti-Ro52/TRIM21 has reduced anti-Ro52/TRIM21 and rheumatoid factor titres.* *• Isolated anti-Ro52/TRIM21 is associated with anaemia and malignancies.*

**Supplementary Information:**

The online version contains supplementary material available at 10.1007/s10067-022-06299-5.

## Introduction

An enigmatic autoantibody, anti-Ro52/tripartite motif-containing protein 21 (TRIM21) IgG targets an intracellular 52 kDa protein involved in the anti-viral response by regulating interferon responses and acting as a cytoplasmic Fc receptor [1]. The autoantibody is not disease-specific and may be found in a range of systemic autoimmune diseases (SADs) such as Sjögren’s syndrome (SS) and organ-specific autoimmune disorders, such as primary biliary cirrhosis (PBC) [2]. Nevertheless, anti-Ro52/TRIM21 IgG has clinical utility and may be a useful prognostic marker of severity in a number of SADs such inflammatory myositides and systemic sclerosis [3, 4].

Anti-Ro52/TRIM21 frequently exists in conjunction with anti-Ro60 and/or anti-La autoantibodies but can be detected in the absence of Ro60/La reactivity (isolated anti-Ro52/TRIM21). Ro60 is an approximately 60 kDa protein that binds non-coding RNAs and participates in RNA quality control within cells [5]. Anti-Ro60 IgG may be found in a number of SADs including SS and is a commonly detected autoantibody in the diagnostic laboratory. La is a 48 kDa autoantigen that binds newly created RNAs to facilitate processing and maturation [6]. Ro60 and La autoantigens form a ribonucleoprotein complex that transiently associates with Ro52/TRIM21 [7], which may explain why autoantibodies against all three proteins commonly co-exist. Intermolecular epitope spreading has been demonstrated in mice immunised with Ro52/TRIM21 protein that subsequently develop autoantibodies to Ro60 and La autoantigens after a delay [8]. However, some patients retain a limited anti-Ro52/TRIM21 response and do not epitope spread within the Ro/La ribonucleoprotein autoantigen system. The absence of anti-Ro60/La autoantibodies in these patients may relate to immunogenetic factors, tendencies (or not) to autoimmunise, and/or the nature of the initiating antigen. Nevertheless, anti-Ro52/TRIM21 is prevalent in a wide range of pathologies [2].

The Ro52/TRIM21 autoantigen is predominantly found in the cytoplasm of nucleated cells, although the HEp-2 antinuclear antibody (ANA) substrate does not reliably identify the IgG autoantibody. Furthermore, anti-Ro52/TRIM21 does not always precipitate using immunoprecipitation assays [9], making it a challenge to identify the autoantibody in the diagnostic laboratory. The most widely used assays to identify anti-Ro52/TRIM21 are the line immunoassay (LIA) and enzyme-linked immunosorbent assay (ELISA). Early studies did not delineate anti-Ro52/TRIM21 from anti-Ro60 and, instead, grouped this under the common “anti-Ro” or “SSA” autoantibody [10]. However, anti-Ro52/TRIM21 co-exists with anti-Ro60 in 50% of cases [11], and there is no cross-reactivity between anti-Ro52/TRIM21 IgG and the Ro60 or La autoantigens [8], suggesting there is value to delineate these autoreactivities.

The aim of this study was to determine whether patients with anti-Ro52/TRIM21 IgG in the absence of anti-Ro60/La have distinct clinical and laboratory associations compared to patients seropositive for Ro52/TRIM21 and Ro60/La. We performed a retrospective, cross-sectional analysis of 346 patients with a range of autoimmune and non-immunological conditions that tested positive for anti-Ro52/TRIM21 within in a 12-month period at a single site.

## Methods

### Data collection

This was a retrospective study performed at the Immunopathology Laboratory, Institute for Clinical Pathology and Medical Research, Westmead Hospital—a major quarternary hospital of Sydney that serves metropolitan and rural areas of New South Wales. Data over a 12-month period from 2020 to 2021 were included. Patients whose serum was referred to our laboratory for anti-extractable nuclear antigen (ENA) testing were screened using an ELISA technique and further characterised by LIA (Euroimmun, Lubeck, Germany) as previously described [12]. Anti-Ro52/TRIM21, anti-Ro60, anti-La and other anti-ENA specificities were defined as positive/detected if the line density reading exceed 10 units, as per manufacturer’s recommendations.

Associated demographic and laboratory data were obtained from the same episode of anti-Ro52/TRIM21 testing. Anti-ENA was defined as the presence of another antibody specificity identified on the LIA other than anti-Ro60 or anti-La. Anti-double stranded DNA (dsDNA) was performed using a chemiluminescent assay and was defined as positive when the value exceeded the manufacturer’s reference range of 0–27 IU/mL. Hypergammaglobulinaemia was defined as an IgG (measured by nephelometry) greater than 16.0 g/L. Anaemia was defined as a haemoglobin < 115 g/L for females and < 135 g/L for males; thrombocytopaenia as a platelet count < 150 × 10^9^ cells/L; neutropaenia as neutrophils < 2.0 × 10^9^ cells/L; and lymphopaenia as lymphocytes < 1.0 × 10^9^ cells/L.

### Statistics

Means of continuous variables were compared using the Student’s *t* test. Categorical variables were analysed using Fisher’s exact test. SPSS statistical package was used in the data analysis. *P* values < 0.05 were considered significant.

### Ethics

Ethics approval for this research was granted by the Western Sydney Local Health District Human Research Ethics Committee.

## Results

Over a 12-month period, we identified 346 individuals who tested positive on LIA for anti-Ro52/TRIM21. The mean age was 57.7 ± 17.3 years and 268 (77.5%) patients were female. Forty percent of the anti-Ro52/TRIM21-positive patients (138/346) did not have anti-Ro60/La autoantibodies, whilst 116 (33.5%) were anti-Ro60 positive, and for 92 (26.6%) patients, almost all patients were triple positive for anti-Ro52/TRIM21, anti-Ro60, and anti-La. A single patient only had autoantibodies to Ro52/TRIM21 and La.

Laboratory records were perused for each patient and relevant data extracted. These were stratified according to presence or absence of anti-Ro60/La in anti-Ro52/TRIM21 patients (Table 1). Patients with isolated anti-Ro52/TRIM21 (no anti-Ro60/La IgG) tended to be older, contain a higher proportion of males, and have lower anti-Ro52/TRIM21 titres, lower rheumatoid factors, lower anti-double stranded DNA positivity, and less neutropaenia compared to patients who were positive for anti-Ro52/TRIM21 and anti-Ro60/La (Table 1). Moreover, isolated anti-Ro52/TRIM21 had higher rates of anaemia (Table 1). To examine the effects of the autoantibodies in the Ro/La autoantigen system, we stratified these parameters according to anti-Ro60 and anti-La positivity (Supplementary Table 1). Many of the differences observed in Table 1 were preserved with the addition of greater proportions of hypergammaglobulinaemia and lymphopaenia in the anti-Ro52/TRIM21^+^Ro60^+^La^+^ group (Supplementary Table 1). The single anti-Ro52^+^Ro60^-^La^+^ patient was excluded from this analysis.

**Table 1:** Demographic and laboratory features of anti-Ro52/TRIM21 subsets. Data is presented as mean ± standard deviation (SD) where applicable. Bolded *P* values represent significant values < 0.05. *ENA*, extractable nuclear antigens. *ESR*, erythrocyte sedimentation rate. *CRP*, C-reactive protein

	Isolated anti-Ro52	Anti-Ro52 with anti-Ro60/La	*P* value
Age (years ± SD)	61.3 ± 16.9	55.3 ± 17.3	**0.002**
Female (*n*, %)	95 / 138 (69)	173 / 208 (83)	**0.002**
Anti-Ro52/TRIM21 densitometry (units ± SD)	68.3 ± 32.2	82.9 ± 25.6	**< 0.001**
Other anti-ENA (mean ± SD)	0.61 ± 0.85	0.58 ± 0.91	0.741
Rheumatoid factor (IU/mL ± SD)	11.0 ± 13.1	35.4 ± 54.3	**< 0.001**
C3 complement ± SD (g/L)	1.17 ± 0.35	1.11 ± 0.29	0.276
C4 complement ± SD (g/L)	0.26 ± 0.21	0.23 ± 0.09	0.253
Positive anti-dsDNA	7 / 67 (10)	31 / 131 (24)	**0.035**
Hypergammaglobulinaemia	21 / 53 (40)	43 / 106 (41)	1.000
ESR ± SD (mm/hr)	29.0 ± 29.6	31.1 ± 24.8	0.633
CRP ± SD (mg/L)	21.9 ± 41.2	15.2 ± 36.8	0.211
Anaemia (*n*, %)	54 / 132 (41)	51 / 205 (25)	**0.003**
Thrombocytopaenia (*n*, %)	22 / 132 (17)	23 / 204 (11)	0.189
Neutropaenia (*n*, %)	7 / 132 (5)	36 / 205 (18)	**< 0.001**
Lymphopaenia (*n*, %)	31 / 132 (23)	55 / 205 (27)	0.524

Next, we questioned whether isolated anti-Ro52/TRIM21 patients differed in their clinical diagnoses compared to their anti-Ro52/TRIM21^+^Ro60^+^La^+^ counterparts. To that end, we interrogated relevant clinical notes and medical records for the patients submitted for analyses. A total of 327 patients (94.5%) of patients had adequate notes to ascertain clinical diagnoses. The most common diagnoses among the patients were SS (21.4%) and SLE (18.7%). Across the organ-specific and systemic immunological disorders, only SLE and SS showed a significantly higher proportion in anti-Ro52/TRIM21^+^Ro60^+^La^+^ group (Table 2).

**Table 2:** Diagnostic correlations with anti-Ro52/TRIM21 specificity. Data is represented as *n* (%). Bolded *P* values represent significant values < 0.05. Underlined entries represent combined statistics for the main diagnostic groups (immunological disorders and miscellaneous diagnoses. *ITP*, immune thrombocytopaenia purpura

	Isolated anti-Ro52	Anti-Ro52 with anti-Ro60/La	*P* value
	(*n* = 131 valid)	(*n* = 196 valid)	
Immunological disorders	50 (38)	137 (70)	**< 0.001**
Autoimmune hepatitis	2 (2)	0 (0)	0.160
Antiphospholipid syndrome	1 (1)	0 (0)	0.401
Cutaneous lupus	0 (0)	3 (2)	0.063
Drug-induced lupus	0 (0)	1 (1)	1.000
Inflammatory myositides	4 (3)	1 (1)	0.162
Mixed connective tissue disease (CTD)	0 (0)	2 (1)	0.518
Neonatal lupus	0 (0)	1 (1)	1.000
Overlap syndromes	1 (1)	4 (2)	0.652
Primary biliary cirrhosis	2 (2)	1 (1)	0.567
Polymyalgia rheumatica	1 (1)	0 (0)	0.401
Rheumatoid arthritis	5 (4)	9 (5)	0.789
Sjögren’s syndrome	7 (5)	63 (32)	**< 0.001**
Systemic lupus erythematosus	16 (12)	45 (23)	**0.014**
Systemic sclerosis	5 (4)	3 (2)	0.275
Undifferentiated CTD	5 (4)	2 (1)	0.121
Vasculitis	1 (1)	2 (1)	1.000
Haematological (e.g. ITP, cytopaenias)	9 (7)	12 (6)	0.821
Malignancies	14 (11)	3 (2)	**0.001**
Neurological syndromes (e.g. autoimmune encephalitis, young strokes)	19 (15)	11 (6)	**0.010**
Renal syndromes (e.g. acute kidney injury)	6 (5)	7 (4)	0.774
Respiratory diagnoses (e.g. interstitial lung disease)	16 (12)	10 (5)	**0.023**
Miscellaneous diagnoses (nil CTD)	17 (13)	16 (8)	0.190
Cardiac (e.g. pericarditis)	7 (5)	3 (2)	0.096
Dermatological disorders	1 (1)	2 (1)	1.000
Hepatitis	0 (0)	1 (1)	1.000
Infections and sepsis	3 (2)	0 (0)	0.063
Thyroid disease	2 (2)	1 (1)	0.567
Urological disease	0 (0)	1 (1)	1.000
Vascular disease	1 (1)	1 (1)	1.000
Others	3 (2)	7 (4)	0.745

For other diagnoses (in the absence of a known organ-specific or systemic immunological disorder), there were higher proportions of patients with isolated anti-Ro52/TRIM21 who had malignancies, neurological disorders and respiratory diseases (Table 2). Supplementary Table 2 lists the exact diagnoses of these patients. As expected, patients positive for anti-Ro52/TRIM21 with anti-Ro/La tended to have a higher proportion of immunological diagnoses than those with isolated anti-Ro52/TRIM21 (129/178, 72.5% vs. 58/118 49.2%, *p* < 0.001). Patients with no apparent immunological diagnosis (miscellaneous diagnoses other than specifically listed in Table 2) comprised 10.1% of the patients (33/327), and the distribution of diagnoses across the two groups did not differ significantly (Table 2).

To determine whether the significant differences in laboratory findings reported in Table 1 were skewed by reduced diagnoses of SS and SLE in the isolated anti-Ro52/TRIM21 group, we conducted further analyses excluding patients with SS or SLE. All significant and non-significant differences in laboratory findings in Table 1 were maintained (data not shown) apart from the sex distribution (with no significant difference between the proportion of females for isolated anti-Ro52/TRIM21 versus anti-Ro52/TRIM21^+^Ro60^+^La^+^ patients [64.7% vs. 71.4%, *p* = 0.317]) and anti-dsDNA positivity (7.0% vs. 12.9%, *p* = 0.381). These data support our hypothesis that isolated anti-Ro52/TRIM21 represent a distinct subgroup of patients with individual laboratory characteristics.

## Discussion

The evaluation of 346 anti-Ro52/TRIM21-positive individuals in our study revealed different clinical and laboratory features associated with the presence or absence of anti-Ro60/La autoantibodies. These findings suggest distinct serological subsets of anti-Ro52/TRIM21 autoantibodies are present in a wide range of immunological and non-immunological disorders (Table 2). Patients with anti-Ro52/TRIM21 in the absence of anti-Ro60/La tended to be older, with reduced rheumatoid factor positivity, anti-dsDNA positivity, and neutropaenia when compared to their anti-Ro52/TRIM21^+^Ro60^+^La^+^ counterparts (Table 1). Those with isolated anti-Ro52/TRIM21 also had lower titres of anti-Ro52/TRIM21 compared to anti-Ro52/TRIM21^+^Ro60^+^La^+^ patients; however, the clinical significance of this relatively small difference is not clear. Moreover, there was a broad variation of anti-Ro52/TRIM21 titres across the groups (as indicated by the standard deviations). Surprisingly, we found higher proportions of patients with anaemia in the isolated anti-Ro52/TRIM21 group. The reason for the latter is also unclear. A previous study demonstrated anti-Ro52/TRIM21 was associated with anaemia in patients with SS [13]. Our study indicates that this association is not limited to SS and occurs regardless of diagnosis.

Our observations indicate that the anti-Ro52/TRIM21^+^Ro60^+^La^+^ group represent a distinct subset of patients defined by intermolecular epitope spreading within the Ro/La ribonucleoprotein autoantigen system. Whether the specificities to Ro60 and La developed temporally after reactivity to Ro52/TRIM21 is unknown. Certainly, anti-Ro52/TRIM21 is one of the earliest autoantibodies that emerges years before the onset of disease in systemic sclerosis [14] giving rise to the possibility that Ro52/TRIM21 may be an inciting autoantigen. However, both anti-Ro52/TRIM21 and anti-Ro60 autoantibodies arise at early timepoints in patients evaluated years prior to diagnosis of SS [15], and patients with isolated anti-Ro60 autoantibodies are also observed [16]. Hence, it is likely that anti-Ro52/TRIM21 and anti-Ro60 are similarly involved in an early autoimmune response, with anti-La emerging later. Immunogenetic influences also dictate which patients may epitope spread since certain human leukocyte antigen (HLA) classes may present peptides more efficiently to autoreactive T cells. HLA-DR3 is highly associated with epitope spreading to the La autoantigen, for example [17].

We unearthed a variety of clinical associations with isolated anti-Ro52/TRIM21, including non-SAD diagnoses. It is possible that although these patients did not have a known SAD at the time of evaluation, and the presence of autoantibodies may herald a future diagnosis, since autoantibodies are known to sometimes predate clinical symptoms and a formal diagnosis [18]. It is also possible that these patients display serological markers of failed immunological tolerance yet never develop any overt clinical autoimmunity in their lifetime. The isolated anti-Ro52/TRIM21 group tended to be older compared to the anti-Ro52/TRIM21 combined with anti-Ro60 and/or -La (Table 1), so it is surprising that additional time did not manifest in proportionally more clinical autoimmunity (Table 2). This is likely due to isolated anti-Ro52/TRIM21 being associated with a variety of immunological and non-immunological conditions compared to anti-Ro60 and anti-La being more specific for autoimmunity. Indeed, another study similarly found a lower proportion of autoimmunity in patients with isolated anti-Ro52/TRIM21 [19]. Nevertheless, SADs like SS and SLE may occasionally have median age of onsets in the fifth and sixth decades [20, 21], giving value for the longitudinal follow-up of these patients.

We have established links with neurological syndromes—specifically, Guillain-Barré syndrome (GBS) that may be a prelude to a later-diagnosed SAD (Supplementary Table 2). In one case report, isolated anti-Ro52/TRIM21 autoantibodies coincided with a diagnoses of GBS in a patient who was later diagnosed with SLE [22]. We also found a higher proportion of isolated anti-Ro52/TRIM21 in patients with malignancy, consistent with previous studies [23, 24]. Tumours have been known to over-express Ro52/TRIM21 [25], being a source of neo-antigens for antibodies to develop. Comparison of patients with SAD- and malignancy-associated anti-Ro52/TRIM21 show differences in Ro52/TRIM21 epitope recognition [23], suggesting possible differences in origin and development of this autoantibody.

Our findings are somewhat congruent with an earlier study [16] that found isolated anti-Ro52/TRIM21 in a similar percentage of laboratory patients. However, the proportion of systemic autoimmunity in our isolated anti-Ro52/TRIM21 subset is lower (Table 2) than some other studies [11, 26, 27]. Possible reasons for this include our specific distinction of patients with other organ-specific autoimmunity, such as autoimmune encephalitis, as a neurological category. In addition, the shorter follow-up period compared to other studies [27] means some patients may not have time to “evolve” into a distinct autoimmune disease. Nevertheless, an important strength of our study is finding unique laboratory profiles of anti-Ro52/TRIM21 patients, independent of SS, and SLE diagnoses, as these have not yet been explored in similar studies. In addition, we also include anti-La IgG in our analysis (Supplementary Table 1) to explore the clinical and laboratory features of patients with intermolecular spreading within the Ro/La ribonucleoprotein complex.

Although the establishment of laboratory features independent to clinical diagnosis may limit the application of our results to the clinical setting, it does, however, provide unique insights into the immunological and laboratory perturbations associated with these autoantibody profiles. Longitudinal analysis of patients with isolated anti-Ro52/TRIM21 may be useful to establish whether this serological profile represents epiphenomena from a dysregulated immune response or is an predictive biomarker for autoimmune disease. In addition, knowing the autoantibody profile of patients may help to predict the possible clinical syndromes that can arise and make clinicians more suspicious for the presence of interstitial lung disease, for instance, when isolated anti-Ro52/TRIM21 exists.

Stereotypic germline antibodies specific for Ro52/TRIM21 may, in part, explain the diverse presence of anti-Ro52/TRIM21 in a wide range of pathologies [28, 29]. A variety of triggers—from infectious to malignant—may cause the convergence of the immunological response to the selection of stereotyped B cells that have evaded immune tolerance checkpoints [30]. Recent interest in the role of the microbiome and molecular mimicry may help explain the emergence of stereotyped autoantibodies and associated SADs [31]. Indeed, the further exploration of anti-Ro52/TRIM21 through epitope and proteomic profiling may help shed light on the origins and progression of the autoantibody and be an avenue for advanced diagnostic and therapeutic strategies [32, 33].

An important limitation to this study was the retrospective and cross-sectional nature of analysis. Autoantibodies are dynamic and we recently established that reactivities to Ro52/TRIM21, Ro60, and La vary widely over time [12], potentially changing anti-Ro52/TRIM21 categories. It would be important, in the future, to conduct longitudinal follow-up studies to check for evolution of symptoms, emergence of clinical diagnoses, and serostatuses of patients. Finally, some of the lack of differences in the diagnoses (high *p* values) in the two groups of patients (Table 2) may result from the low numbers of patients in subgroups (type II error). This may reflect the natural low numbers of patients found and ideally should be examined in future, larger studies. This is particularly pronounced in the myositis and PBC groups where isolated anti-Ro52/TRIM21 is more commonly found [19]; yet, we failed to detect any significant differences.

To conclude, stratification of patients by anti-Ro52/TRIM21 seroreactivity have revealed interesting clinical and laboratory associations. Anti-Ro52/TRIM21 with anti-Ro60/La patients tend to be younger, female, more immunologically active and associated with clinical diagnoses such as SLE. Our findings, therefore, support accurate and separate detection of anti-Ro52/TRIM21 from anti-Ro60 which allows stratification and prognostication of patients.

## Supplementary Information

ESM 1 (DOCX 18.2 KB)
