# Supplementary material for: Anti-Ro52/TRIM21 serological subsets identify differential clinical and laboratory parameters
Source: Clin Rheumatol. 2022 Jul 23;41(11):3495–501. doi: 10.1007/s10067-022-06299-5 (PMC9568495; doi:10.1007/s10067-022-06299-5)
Supplement: Supplementary file 1 — (DOCX 18.2 KB) [file 10067_2022_6299_MOESM1_ESM.docx]

**Supplementary Table 1. Demographic and laboratory features of anti-Ro52/TRIM21 subsets, stratified by anti-Ro60 and anti-La positivity.** Column statistics for the Anti-Ro52^+^Ro60^+^La^-^ and Anti-Ro52^+^Ro60^+^La^+^ were compared to the isolated anti-Ro52/TRIM21 group (first column). * *p* < 0.05; ** *p* < 0.01; *** *p* < 0.001.

|  | **Anti-Ro52^+^Ro60^-^La^-^**  **(*n* = 138)** | **Anti-Ro52^+^Ro60^+^**  **La^-^**  **(*n* = 115)** | **Anti-Ro52^+^Ro60^+^La^+^**  **(*n* = 97)** |
| --- | --- | --- | --- |
| Age (years ± SD) | 61.3 ± 16.9 | 54.6 ± 17.2** | 56.1 ± 17.6* |
| Female (n, %) | 95 / 138 (69) | 100 / 115 (87)** | 72 / 92 (78) |
| Anti-Ro52/TRIM21 densitometry (units ± SD) | 68.3 ± 32.2 | 75.6 ± 27.4 | 92.0 ± 20.0*** |
| Average other anti-ENA (n ± SD) | 0.61 ± 0.85 | 0.58 ± 0.98 | 0.58 ± 0.82 |
| Rheumatoid factor (IU/mL ± SD) | 11.0 ± 13.1 | 30.5 ± 58.4** | 42.4 ± 48.8*** |
| C3 ± SD complement (g/L) | 1.17 ± 0.35 | 1.18 ± 0.32 | 1.11 ± 0.23 |
| C4 ± SD complement (g/L) | 0.26 ± 0.21 | 0.24 ± 0.10 | 0.22 ± 0.08 |
| Positive anti-dsDNA | 7 / 67 (10) | 19 / 76 (25)* | 12 / 55 (22) |
| Hypergammaglobulinaemia | 21 / 53 (40) | 14 / 57 (25) | 29 / 48 (60)* |
| ESR ± SD (mm/hr) | 29.0 ± 29.6 | 26.6 ± 21.3 | 36.4 ± 27.7 |
| CRP ± SD (mg/L) | 21.9 ± 41.2 | 15.4 ± 40.0 | 15.2 ± 32.8 |
| Anaemia (n, %) | 54 / 132 (41) | 23 / 114 (20)** | 28 / 90 (31) |
| Thrombocytopaenia (n, %) | 22 / 132 (17) | 14 / 114 (12) | 9 / 89 (10) |
| Neutropaenia (n, %) | 7 / 132 (5) | 16 / 114 (14)* | 19 / 90 (21)*** |
| Lymphopaenia (n, %) | 31 / 132 (23) | 30 / 114 (26) | 29 / 48 (60)* |

**Supplementary Table 2. Tabulated diagnoses of patients associated with anti-Ro52/TRIM21.**

| **Malignancies** | **Neurological Syndromes** | **Respiratory Diagnoses** |
| --- | --- | --- |
| Renal cell carcinoma | Peripheral neuropathy (x5) | Pulmonary infiltrates |
| Multiple myeloma | Young stroke (x4) | Asthma (x4) |
| Neuroendocrine tumour | Limb weakness | Pulmonary hypertension (x3) |
| Cholangiocarcinoma (x2) | Guillain-Barré syndrome (x5) | Bronchiectasis (x2) |
| Atrial spindle cell neoplasm | Migraines / headache (x3) | Respiratory failure |
| Lung cancer | Miller-Fisher syndrome | Pulmonary fibrosis / interstitial lung disease (x10) |
| Malignancy of unknown primary | Cerebellar syndrome | Pleural effusion / pleuritis for investigation (x2) |
| Breast cancer (x3) | Cranial nerve palsy (x2) | Organising pneumonia (x2) |
| Ovarian cancer (x2) | Encephalitis (x5) | Haemoptysis |
| Diffuse large B cell lymphoma | Cramer-Niederdellmann syndrome |  |
| Colorectal cancer | Refractory epilepsy (x2) |  |
| Hepatocellular carcinoma |  |  |
| Melanoma |  |  |
